# Mobile reminders to improve opportunistic screening of type 2 diabetes mellitus: Data documentation and data analysis plan of a randomized trial data

**DOI:** 10.1016/j.dib.2016.01.037

**Published:** 2016-01-29

**Authors:** Hemant Deepak Shewade, Sathish Kumar

**Affiliations:** a International Union Against Tuberculosis and Lung Disease (The Union), South-East Asia Office, New Delhi, India; b Indira Gandhi Medical College and Research Institute (IGMCRI), Puducherry, India

**Keywords:** Randomized controlled trial, Diabetes mellitus, type 2, Reminder system, Opportunistic screening, Loss to follow up, Data set

## Abstract

This Data in Brief article contains individual level data of a randomized trial in a primary care setting. This trial offered mobile reminder to follow up for definitive tests during opportunistic screening of diabetes mellitus in Puducherry, India (2014). (“Effect of mobile reminders on screening yield during opportunistic screening for type 2 diabetes mellitus in a primary health care setting: a randomized trial” (Kumar et al., 2015) [1]) Variables collected included the baseline characteristics of study participants (*n*=390) and information on initial screening and eligibility for definitive test, study group (intervention/control), follow up for definitive test and definitive test results. The data was double entered with adequate checks and validated in EpiData. Final data after correcting the data entry errors has been shared here. In addition, we have shared data entry plan, EpiData triplet files for data entry and program file for data analysis. They may be used by other researchers who intend to replicate this research in their setting.

## Specifications Table

**Table:** 

Subject area	*Medicine*
More specific subject area	*Community medicine*
Type of data	*Text (ASCII) files*
How data was acquired	*Data pertaining to routine opportunistic screening in the PHC was collected. A mobile reminder was added as an intervention*
Data format	*Raw data in EpiData (.rec) format*
Experimental factors	*Study participants eligible for definitive tests were randomized (central randomization) to intervention and control arm after the OPD. Those in intervention arm received a standard reminder over mobile phone to follow up*.
Experimental features	*Case record form of study participants and blood glucose measurements*
Data source location	*Puducherry, India*
Data accessibility	*Data is in this article*

## Value of the data

• This dataset demonstrates how a pragmatic RCT conducted in operational settings with limited number of variables can yield the desired results.
• The case record plan, data entry plan, data structure, program file for analysis may be used by other researchers who intend to replicate this research in their setting.
• We may consider collaborating with researchers who want to replicate a similar study in their setting: support to restructure this electronic data collection tool and program file for analysis to suit their setting.

## 1 Data

In this dataset, the variables collected for each study participant (*n*=390) were patient id, age, sex, random blood glucose (RBG). Among those who were eligible for definitive tests, the variables collected were group (intervention/control), whether the mobile reminder call was answered (for intervention arm), follow up done (yes/no), fasting and post-prandial blood glucose (for those who followed up). Some variables were derived during data entry: eligibility for definitive tests based on RBG value and diabetes status based on the fasting and post-prandial blood glucose values. Additional variables were derived during data analysis: epidemiologic diagnosis of diabetes and pre-diabetes (if either one of the two definitive tests was within the diabetic or pre-diabetic range).

We are also sharing the case record form, data entry plan (codebook), empty EpiData files, EpiData files which include the REC file containing data and EpiData program file (computer code) for analysis (EpiData version 3.1 for entry and version 2.2.2.182 for analysis, EpiData Association, Odense, Denmark). EpiData triplet files include questionnaire (QES), record (REC), and check (CHK) files. *Q*u*ES*tionnaire file defines the structure of the database and layout of data entry. *REC*ord file holds the entered data and into which data is entered. *CH*ec*K* file holds the data checking rules during data entry. These EpiData triplet files are simple text (ASCII) files [2].

## 2 Experimental design, materials and methods

The study was approved by the Institute Ethics Committee, Indira Gandhi Medical College and Research Institute, Puducherry. The study was registered with Clinical Trials Registry – India (CTRI/2014/10/005138). The data collection was carried out in accordance with The Code of Ethics of the World Medical Association (Declaration of Helsinki).

This was a randomized trial conducted in a primary health care setting offering routine opportunistic screening for diabetes mellitus [1]. As the patients registered for outpatient department visit, patients satisfying the selection criteria were given the option to be part of the study. After getting written informed consent, patient id was given; and mobile phone number, age and sex recorded. RBG was done using a glucometer and data recorded. Those with RBG ≥6.1 mmol/l (eligible outpatients) were given a slip for definitive tests and asked to return in overnight (8 h) fasting state. After outpatient department visit, eligible outpatients were randomized into intervention and control arm by an independent statistician (central randomization). Investigator made a standard call over mobile phone the same evening to the intervention arm. A note was made of those participants in intervention arm who did not attend the call. Data of FBG and PPBG among those who returned for follow up was noted by the laboratory technician at the laboratory. Patient id was used to link the data of patient at registration and at the laboratory. Data was collected in a case record form.

A codebook or plan of data entry was prepared using a Microsoft Excel sheet before data entry. With the codebook as the base, EpiData triplet files were created for electronic data entry. A program file was prepared for data analysis and can be run using EpiData analysis software.

## Funding statement

This was an operational research carried out using available resources and manpower at the Primary Health Centre. The authors declare that there was no source of funding separately for the research.

**Conflict of interest**

None declared.
