# Supplementary material for: Mobile reminders to improve opportunistic screening of type 2 diabetes mellitus: Data documentation and data analysis plan of a randomized trial data
Source: Data Brief. 2016 Jan 29;6:817–9. doi: 10.1016/j.dib.2016.01.037 (PMC4749946; doi:10.1016/j.dib.2016.01.037)
Supplement: Supplementary file 2 — Supplementary material [file mmc2.zip › Supplementary material_Codebook_qes rec chk triplet_program file/1. Case record form and Codebook_Plan for data entry/Case record form.docx]

| Patient ID |  |  |  |  |  |  |  |
| --- | --- | --- | --- | --- | --- | --- | --- |
| Name |  |  |  |  |  |  |  |
| Mobile Number |  |  |  |  |  |  |  |
| Age in completed years |  |  |  |  |  |  |  |
| Sex |  |  |  |  |  |  |  |
| Random blood sugar |  |  |  |  |  |  |  |
| Eligibility for definitive test |  |  |  |  |  |  |  |
| Study arm (Intervention / control) |  |  |  |  |  |  |  |
| Whether call was answered  (for Intervention arm) |  |  |  |  |  |  |  |
| Whether follow up was done |  |  |  |  |  |  |  |
| Fasting blood glucose  (if follow up was done) |  |  |  |  |  |  |  |
| Postprandial blood glucose  (if follow up was done) |  |  |  |  |  |  |  |
